# Bradykinin and Galectin-3 in Survived and Deceased Patients with COVID-19 Pneumonia: An Increasingly Promising Biochemical Target

**DOI:** 10.1155/2022/7920915

**Published:** 2022-10-27

**Authors:** Tamara Nikolic Turnic, Viseslav Popadic, Slobodan Klasnja, Ana Sekulic, Novica Nikolic, Vladimir Zivkovic, Nevena Jeremic, Marijana Andjic, Nevena Draginic, Ivan Srejovic, Jovana Jeremic, Marija Zdravkovic, Vladimir Jakovljevic

**Affiliations:** ^1^Department of Pharmacy, Faculty of Medical Sciences, University of Kragujevac, Serbia; ^2^N.A.Semashko Public Health and Healthcare Department, F.F. Erismann Institute of Public Health, I.M. Sechenov First Moscow State Medical University (Sechenov University), Moscow, Russia; ^3^University Clinical Hospital Center Bežanijska kosa, Belgrade, Serbia; ^4^Department of Physiology, Faculty of Medical Sciences, University of Kragujevac, Serbia; ^5^Department of Pharmacology, 1st Moscow State Medical, University IM Sechenov, Trubetskaya street 8, str. 2, 119991 Moscow, Russia; ^6^I.M. Shechenov First Moscow State Medical University (Sechenov University), 8-2 Trubetskaya st., Moscow, Russia; ^7^Faculty of Medicine, University of Belgrade, Belgrade, Serbia; ^8^Department of Human Pathology, 1st Moscow State Medical, University IM Sechenov, Trubetskaya street 8, str. 2, 119991 Moscow, Russia

## Abstract

**Introduction:**

There are still no definite curative or preventive strategies for COVID-19 disease. It is crucial to fully comprehend the pathogenesis of COVID-19 infection so that we can develop expedient pharmacological protocols. While the impact of cytokine storm on COVID-19 severity has been one of the most tested hypotheses, the role of bradykinin and various other oxidative stress markers has been relatively under-researched. Their levels can be determined immediately after a hospital admission so they could be used as early predictors of the further development of the disease.

**Aim:**

The study aims at evaluating the possibility of using bradykinin and galectin-3 levels as early predictors that COVID-19 disease will progress into a severe case. *Material and methods*. The study was conducted as a prospective cross-sectional study. It included 47 consecutive adult patients with confirmed SARS-CoV-2 infection and COVID-19 pneumonia. All study subjects were admitted for a hospital treatment to the tertiary Clinical Hospital Center Bezanijska kosa, Belgrade, Serbia on June 2021. The blood samples were collected at the patients' admission. The analyses of demographic, radiological, and clinical data were later conducted for both groups (the deceased patients and those who survived). In addition, we analyzed the potential relations between the outcome and the levels of bradykinin and galectin-3 measured immediately after the patients were admitted to the hospital.

**Results:**

The patients who passed away were predominantly older men with comorbidities. We recorded higher CT scores in the deceased patients and the significantly higher levels of urea, creatinine, CK, troponine, CRP, and other laboratory markers. They stayed at the ICU unit longer and required mechanical ventilation more frequently than the patients who survived. On the other hand, no differences were recorded in the time periods passing from the onset of the systems to the hospital admissions. Finally, we can highlight several independent predictors of mortality in patients with COVID-19 pneumonia, including the following: (1) patients who are 50 or more years old, (2) with in-hospital stays are longer that 4 days, (3) bradykinin levels surpass 220000 pg/ml, (4) D-dimer, creatinine, and CRP are elevated, and (5) comorbidities were present (such as hypertension and diabetes).

**Conclusion:**

The present study strongly supports the bradykinin storm hypothesis. Since elevated bradykinin levels have been found in most COVID-19 cases with fatal outcomes, the future therapeutical strategies for COVID-19 have to be focused on reducing bradykinin serum concentrations.

## 1. Introduction

Coronavirus disease (COVID-19), caused by SARS-CoV-2 viral infection, has resulted in more than 583 million infections and more than six million deaths globally [1]. SARS-CoV-2 comprises four crucial structural proteins which are essential for its infectivity and replication: spike (S), membrane (M), envelope (E), and nucleocapsid (N) proteins. The structural specificities are responsible for the diversity of clinical manifestations in infectious diseases [2]. The spectrum of COVID-19 disease ranges from asymptomatic to critical cases [2]. The pathology of COVID-19 is most commonly described as moderate or severe respiratory syndrome. In 81% of the cases, the disease has been classified as mild, and in 14% as severe [2]. In some cases, other clinical features have been reported, such as coagulation disturbances, multiple organ failure, shock, and death [3].

The pathophysiological processes of SARS-CoV-2 infection manifest as disturbances of laboratory markers and deterioration of respiratory functions, but also as venous and arterial thromboembolism [4]. Angiotensin-converting enzyme-2 (ACE-2) with its receptor is the principal enzyme that the virus uses. It is predominantly present, inter alia, in blood vessels, lungs, heart, and kidneys [5].

Bradykinin (BK) is a hypotensive cytokine. It has been mentioned in the previous studies as a potential contributing factor in the pathophysiology of severe COVID-19 disease manifestations [6]. *Bradykinin storm* is an imbalance of bradykinin caused by a dysregulation in kallikrein-kinin system (KKS). Since its discovery in snake venom, bradykinin has been used as an explanation for physiopathological phenomena in inflammatory conditions. BK is a regulator of tissue blood flow and a regulator of vasomotor activity. It is an additional component of the renin-angiotensin system (RAS). On the other hand, in very high concentrations, BK has a prominent role in inflammatory and oxidative process [6].Galectin-3 (beta-galactoside-binding lectin) is described as a marker of lung disease and it has been seen as an important contributor to COVID-19 disease [7]. It plays a significant role in the response of an immune system, i.e., in modulating the lifecycle of immune cells, in angiogenesis, and in reparative lung injuries. During SARS-CoV-2 viral infection, galectin-3 could facilitate viral entrance into hosts' immune cells and it can enhance the production and release of cytokine [8, 9]. Galectin-3, also known as Mac-2, L29, CBP35, and etaBP, is a secreted lectin which participates in antimicrobial immunity via the opsonization of pathogen, the recruitment of macrophage, and the activation of mast cells and neutrophils. It can also contribute to chronic inflammation and fibrosis [7–9].

On the other hand, a host's response is essential by the virtue of immune response and antibody release [10, 11]. SARS-CoV-2 infection has been also shown to cause hypoxemia. This leads to the accumulation of oxygen-free radicals and lactic acid, the changes in intracellular pH and electrolytes, and a further cellular damage [12–16]. We know that different viruses employ different mechanisms to induce redox imbalance and oxidative stress [16]. In COVID-19 disease and respiratory disease, the underlying production of reactive oxygen species (ROS) is expected since all patients with pulmonary diseases are affected by chronic oxidative stress [17]. Pulmonary alveolar macrophages produce ROS in a small amount, but during their higher activity, the production is even more boosted than it usually is. Moreover, immune cells represent a large source of ROS since the activation of nitric oxidase-2 initiates ROS production in viral infections. The viral pneumonia caused by SARS-CoV-2 overactivates an immune response in lungs which then leads to oxidative stress [18, 19].

The connection between oxidative stress and inflammatory response during COVID-19 disease results from the concurrent activity of endothelial cells and immune response. The authors have suggested that the regulation of endothelial function could prevent the cytokine storm [19, 20].

Still, there are no curative or preventive strategies for COVID-19. The deeper understanding of the pathogenesis of a COVID-19 infection is essential for the rational development of pharmacological protocols. Due to their proinflamatory and oxidative effects, it is crucial to investigate the clinical repercussions of BK and Galectin 3 in survived and deceased patients with confirmed COVID-19 pneumonia. Several studies have revealed that the hyperinflammatory response, induced by SARS-CoV-2 infection and acute pneumonia, is a major risk factor for developing the severe forms of the disease that can result in death. Still, there is a lack of evidence on how bradykinin and galectin-3 are involved in the pathogenesis and fatal clinical outcomes of COVID-19 disease. These findings can indicate promising therapeutical targets and independent predictors of patients' chances of survival. The present study aims at investigating the potential usefulness of bradykinin and galectin-3 levels, obtained at hospital admission, as predictors of COVID-19 progression into severe or deadly forms of the disease.

## 2. Materials and Methods

### 2.1. Ethical Concerns

This study was approved by the Local Institutional Committee of the University Clinical Hospital Center Bežanijska kosa, Belgrade, Serbia (reference number 6609). All procedures were conducted in accordance with the Declaration of Helsinki (revision 2013) and Good Clinical Practice. The written informed consents were obtained from all participants before their inclusion in the study. A preprint of this manuscript has been previously published [21].

### 2.2. Study Design

The study was conducted as a prospective cross-sectional study. It included 47 adult patients with confirmed SARS-CoV-2 infection and radiologically-confirmed pneumonia who were admitted to University Clinical Hospital Center Bežanijska kosa, Belgrade, Serbia, in June 2021. The main criteria for the participation in the study in this study included the following: (1) that a patient was 18 or more years old, (2) that COVID-like pneumonia was confirmed radiographically, and (3) that the patients consented to participate. All patients were given adequate supportive care and were administered the proper therapy according to the pharmacological protocols (created by hospital's COVID Management Guidelines Committee and the Ministry of Health of the Republic of Serbia).

### 2.3. Diagnosis of COVID-19 Pneumonia

COVID-19 infection was confirmed with reverse transcriptase-polymerase chain reaction (RT-PCR) from nasopharyngeal swab sample. COVID-19 pneumonia was confirmed radiographically by chest X-ray or chest CT. Chest CT was performed in all participants so that we could evaluate the stage and extensiveness of interstitial pneumonia with more precision. The typical COVID-19 pneumonia changes were evaluated by examining the bilateral lung involvement (with ground-glass opacities), the lung consolidations, the interlobular septal thickenings, the crazy pavings, the pleural effusions, and the lymphadenopathy of our patients. According to the severity of the observed changes, every lobe was given 0–5 points (upper, middle, and lower right lung lobe and upper and lower left lung lobe) forming the maximum possible score of 25 points. Except scoring, the disease was also classified into four stages (early, progressive, peak, and resolutive). The main clinical criteria for the ICU admission was the detected progression of the disease observed through radiographic or chest CT examinations, the peripheral oxygen saturation (Sp02) below 93% (despite maximal conventional supportive oxygen therapy through a nasal cannula, conventional oxygen, or nonrebreather mask), and the further impairment of the results obtained through arterial blood gas tests and laboratory tests (mainly the increases of inflammatory parameters).

### 2.4. Laboratory Data Collection at Hospital Admission

The demographic data, comorbidities, and laboratory parameters were collected from all patients at their admission. The laboratory parameters included a complete hemogram, neutrophile-lymphocyte ratio, blood sugar, serum ferritin, coagulation status and D-dimer, serum lactate dehydrogenase, and troponin; renal function test, liver function test, and electrolyte balance. The treatment details were also recorded, such as the use of steroids, anticoagulants, high-flow nasal cannula, noninvasive, and invasive ventilation.

### 2.5. Systemic Oxidative Stress Markers

The following biomarkers of oxidative stress were measured in plasma samples: superoxide anion radical (O_2_^−^), hydrogen peroxide (H_2_O_2_), nitrite (NO_2_^−^), index of lipid peroxidation (measured as TBARS, i.e. thiobarbituric acid reactive substances), and activity enzymes (catalase (CAT), superoxide-dismutase (SOD), and reduced glutathione (GSH)). All these biochemical parameters of oxidative stress were measured spectrophotometrically (Shimadzu UV-1800 spectrophotometer, Japan, manufacturer number 00182).

The oxygen concentrations were evaluated by following Auclair's method. The NTB (Nitro Blue Tetrazolium) reagent was added to TRIS buffer (assay mixture) and the measurements were performed at the wavelength of 530 nm [22].

The protocol for the quantification of H_2_O_2_ was based on the oxidation of phenol red by H2O2 in the presence of horseradish peroxidase as a catalyst. The levels of H_2_O_2_ were then measured at the wavelength of 610 nm [23].

The TBARS method was used for evaluating the lipid peroxidation indices. The biological specimen (with 1% thiobarbituric acid (TBA) and 0.05 M sodium hydroxide (NaOH)) were heated at 100° C for 15 minutes. Finally, the measurements were performed at 530 nm [24].

The nitric oxide levels were evaluated indirectly, i.e., via measuring the nitrite levels. Green's method, with the Griess reagent (containing 1% sulfanilamide in 5% phosphoric acid/0.1% naphthalene ethylenediamine dihydrochloride) was applied here [25]. The sample was precipitated with 30% sulfosalicylic acid, was vortexed for 30 min, and was then centrifuged at 30009 g. The equal volumes of the supernatant and Griess's reagent were added and incubated for 10 minutes in the dark and then measured at 543 nmol/l.

The antioxidant enzyme CAT quantification was carried out according to McCord's method [26]. We used CAT buffer, prepared lysate sample, and 10 mM of H_2_O_2_. The CAT activity was measured spectrophotometrically at the wavelength of 360 nm and was expressed in nmol/ml plasma.

The SOD activity was evaluated by applying Beutler's epinephrine method [27]. The sample was first mixed with carbonate buffer, and then epinephrine was added to the mixture afterward. The SOD activity was measured at the wavelength of 470 nm and was expressed as U/ml plasma.

The GSH levels were measured by following Beutler's method [28]. The method assumes the reaction of GSH oxidation with 5.5-dithiol-bis-6.2-nitrobenzoic acid. The levels of GSH were measured spectrophotometrically at the wavelength of 420 nm and were expressed in nmol/ml plasma.

### 2.6. Enzyme-Linked Immunosorbent Assay for Bradykinin and Galectin-3 (ELISA)

The serum bradykinin levels were measured with a bradykinin ELISA kit (ab136936, Abcam) by following the manufacturer's instructions. All samples were tested in duplicate. The optical densities (OD) were measured at the wavelength of 450 nm on a microtiter plate reader (UT-2100C, MRC, UK, manufacturer number 452104038IEX).

Ninety-six-well microplates were precoated with capture polyclonal antibody (goat) to human galectin-3 (Human galectin-3 ELISA solid Phase Sandwich Elisa, R&D system, Inc.) and washed three times with wash buffer 1% Tween 20 (Sigma–Aldrich, St. Louis, MO) in PBS. The samples (50 ll) were added in duplicate to each well, which contained 50 ll of the sample diluent. Detection antibody was diluted in reagent diluent and was then added to each well and incubated at room temperature (RT) for 2 hours on a microplate shaker set at 200 rpm. After washing and incubation, the reaction was stopped by a stop solution. The absorbance of each sample was measured at 450 nm in a microtiter plate reader (UT-2100C, MRC, UK, manufacturer number 452104038IEX). A standard curve ranging from 0.156 to 10 ng/ml of galectin-3 was generated for each ELISA.

### 2.7. Secondary Outcomes of Patients with COVID-19 Pneumonia

These data were collected retrospectively from the hospital's electronic medical records by trained staff. An electronic data capture tool was used for these purposes. We recorded the time periods passing from the first symptoms to the patients' admission, the durations of the ICU stays, and the durations of mechanical ventilation for both groups (the survived and deceased patients).

### 2.8. Statistical Analysis

The patients' data were summarized by using standard descriptive statistics, i.e., means for continuous variables and count/percent for categorical variables. The correlation analyses were used to test the potential impact of cytokines and oxidative biomarkers. The laboratory test results were evaluated with the Mann–Whitney *U* test as appropriate, while the patients' data were tested with the Chi Square Test. In addition, the Cox regression model was used to test the relations between cytokine values and patient demographics and comorbidities. The Cox proportional hazards model was employed to estimate the death hazards. It includes covariance adjustment. The covariates (e.g., patient demographics, comorbidities, and laboratory test results) were determined by the backward elimination method. The point estimates (HRs), along with the corresponding 95% CIs, predicted probabilities for survival. We obtained the cumulative incidence curves. The analyses used two-sided tests and were performed in the SPSS version 26.0 for Macintosh.

## 3. Results

Between the August and September of 2021, 47 consecutive patients with confirmed COVID-19 pneumonia were recruited for the study. 31.2% of them passed away. The mean age of the survivors was 46.50 years and the mean age of the deceased patients was 72.53 (Table 1.). The male patients were present in a significantly higher percentage in both groups.

Regarding the presence of comorbidities, a higher percentage of patients with hypertension (93.3%), diabetes (20.0%), and obesity (6.67%) was recorded among the group of the deceased patients. Other comorbidities (such as coronary artery disease, cardiomyopathy, and chronic kidney disease) were also more frequent in the deceased patients. Chronic obstructive pulmonary disease and asthma were not detected in any group (Table 1).

The laboratory blood tests were significantly impacted in both groups (Table 2). The levels of serum creatinine, uremic acid, direct bilirubin, AST, LDH, CK, hsTnT, chloride ions, and C-reactive protein were significantly higher in the deceased patients. The serum concentrations of urea were significantly lower in the survived patients (Table 2).

The significant differences between the survived and deceased patients were detected in the total blood counts and coagulation statuses (Table 3). The deceased patients had the higher neutrophils counts, D-dimer concentrations, and fibrinogen activities. On the other hand, the same group had lower levels of hemoglobin, platelets, lymphocytes, monocytes, and Factor IX and Factor XII activities (Table 3).

The secondary outcomes were also different (Table 4). The deceased patients stay in the hospital longer than the survivors (Table 4). Moreover, their CT scores were worse and they spent more time on the mechanical ventilation and in the ICU. Interestingly, the time periods which had passed from the onset of the first symptoms to the hospital admission were fairly similar for both groups (Table 4).

### 3.1. Redox Status and Bradykinin Storm in Patients with COVID-19 Pneumonia

**Figure 1:**
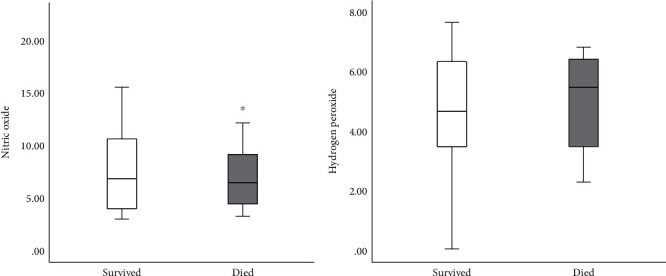
The mean plasma values of nitric oxide (nmol/ml) and hydrogen peroxide (nmol/ml) in survived [*n* = 32] and deceased patients [*n* = 15] at admission. Statistical significance was confirmed by Mann–Whitney *U* test.

**Figure 2:**
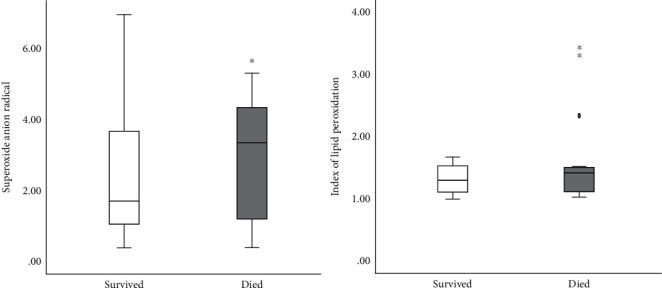
The mean plasma values of superoxide anion radical (nmol/ml) and index of lipid peroxidation measured as TBARS (*μ*mol/ml) in survived [*n* = 32] and deceased patients [*n* = 15] at admission. Statistical significance was confirmed by Mann–Whitney *U* test.

**Figure 3:**
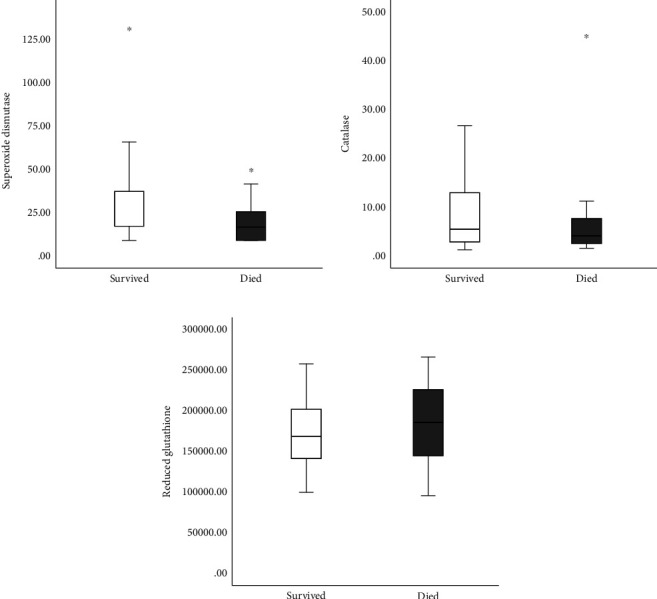
The mean hemolysate activity of superoxide dismutase (U/Hg × 109), catalase (U/Hg × 109), and reduced glutathione (U/Hg × 109) in survived [*n* = 32] and deceased patients [*n* = 15] at admission. Statistical significance was confirmed by Mann–Whitney *U* test.

**Figure 4:**
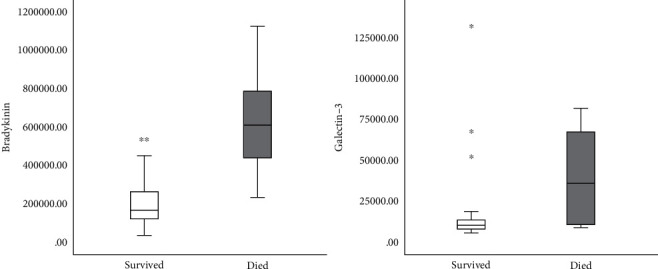
The mean serum concentration of bradykinin (pg/ml) and galectin-3 (ng/ml) in survived [*n* = 32] and deceased patients [*n* = 15] at admission. Statistical significance was confirmed by Mann–Whitney *U* test.

The Figures 1–4 show the mean concentrations of oxidative stress markers, the activities of antioxidant enzymes, and the concentrations of bradykinin and galectin-3 in the survived and deceased patients. We detected significantly lower levels of nitric oxide, and the activity of superoxide dismutase and the higher levels of superoxide anion radical and the index of lipid peroxidation in the deceased patients (Figures 1–3). Bradykinin and galectin-3 concentrations were significantly higher in the deceased patients (Figure 4).

The correlation analysis among all COVID-19 patients confirmed the statistically significant correlation between some markers of oxidative stress and cytokine bradykinin and peptide galectin-3 (Table 5). Serum bradykinin was in a positive weak correlation with the levels of plasma hydrogen peroxide, and in an inverse weak correlation with the activity of superoxide dismutase. Moreover, galectin-3 correlates with the index of lipid peroxidation in a similar manner (Table 5).

**Figure 5:**
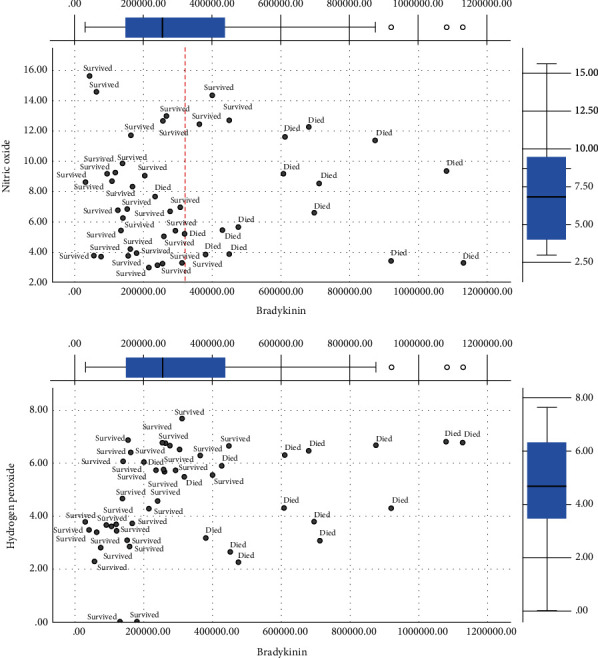
Distribution of survived and deceased patients in different concentrations of nitric oxide/hydrogen peroxide and bradykinin. Red line represents an observed cut-off value of bradykinin [pg/ml] for predicting death in patients with COVID-19 pneumonia.

**Figure 6:**
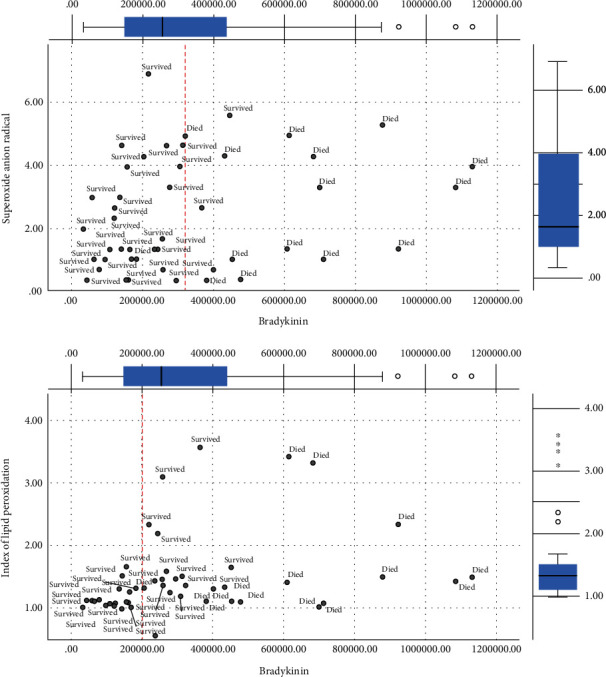
Distribution of survived and deceased patients in different concentrations of superoxide anion radical/index of lipid peroxidation and bradykinin. Red line represents an observed cut-off value of bradykinin [pg/ml] for predicting death in patients with COVID-19 pneumonia.

**Figure 7:**
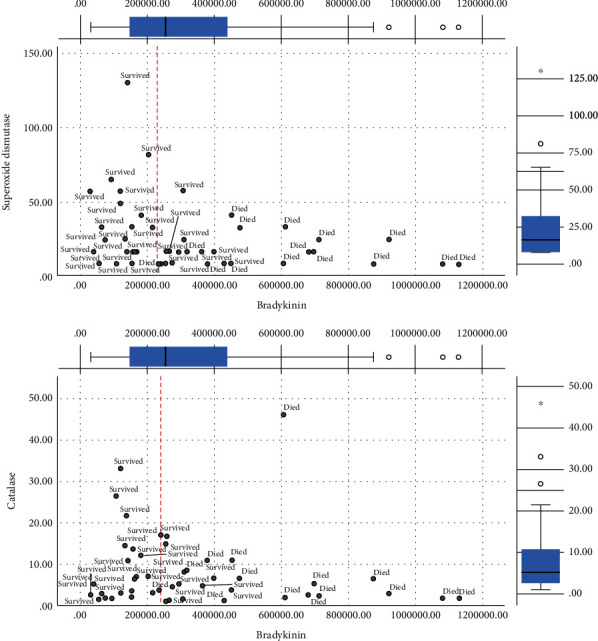
Distribution of survived and deceased patients in different activities of superoxide dismutase/catalase and bradykinin [pg/ml]. Red line represents an observed cut-off value of bradykinin for predicting death in patients with COVID-19 pneumonia.

**Figure 8:**
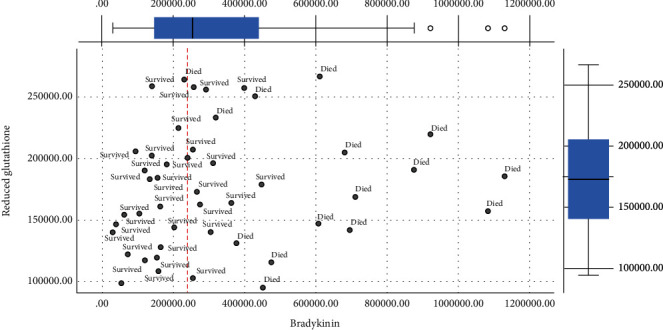
Distribution of survived and deceased patients in different contents of reduced glutathione and bradykinin [pg/ml]. Red line represents an observed cut-off value of bradykinin for predicting death in patients with COVID-19 pneumonia.

Since bradykinin showed a good linear correlation with the detected oxidative stress levels, we evaluated the potential cut-off values of bradykinin, which could be taken as the borderline between the positive and fatal outcomes. Figures 5–8 present the cut-off values of bradykinin (the red line). The levels ranging from 200000 to 280000 pg/ml represent the borderline between the survived and the deceased patients. These values can be used as a significant diagnostic and prognostic sign for changing the therapy protocols and preventing fatal outcomes (Figures 5–8).

The Cox regression analysis reveals the following independent predictors of mortality in the patients suffering from COVID-19 pneumonia: (1) age above 50, (2) in-hospital stays surpassing four days, (3) the bradykinin levels above 220000 pg/ml, (4) the elevated D-dimer, creatinine, and/or CRP, and (5) the presence of comorbidities (hypertension and diabetes) (Table 6).

## 4. Discussion

This was conducted as a prospective cross-sectional study. It included 47 consecutive adult patients with confirmed SARS-CoV-2 infection and COVID-19 pneumonia. All patients fulfilled the criteria for hospital treatment and were admitted to the University Clinical Hospital Center Bežanijska kosa, Belgrade, Serbia, in June 2021. The main criteria for the participation in the study included the following: (1) that a patient was 18 or more years old, (2) that COVID-like pneumonia was confirmed radiographically, and (3) that patients consented to participate. All patients were given adequate supportive care and were administered the proper therapy according to the pharmacological protocols (created by hospital's COVID Management Guidelines Committee and the Ministry of Health of the Republic of Serbia). This study aimed at detecting the major risk factors for developing severe conditions of COVID-19 disease, with special reference to bradykinin and galectin-3. Their levels can be measured immediately after the admission and they might be used as indicators that the disease will progress in undesirable directions. The data regarding the role and mechanisms of bradykinin and galectin-3 in severe COVID-19 cases have not been fully explored. Thus, our findings can provide a valuable insight. They can indicate the therapeutical targets and can be used as independent predictors of patients' survival.

The first part of the study focused on the basic demographic characteristics of the patients. The deceased patients were older than the patients who survived. In both groups, male patients were dominant.

We recorded the higher shares of the patients with hypertension (93.3%), diabetes (20.0%), and obesity (6.67%) among the deceased patients. Other comorbidities, such as coronary disease, cardiomyopathy, and chronic kidney disease were also more frequent in this group. Chronic obstructive pulmonary disease and asthma were not detected in any group (Table 1). The previous studies have recognized the probable and possible risk factors for severe COVID-19, but still there have been some cases with unusual outcomes and prognoses. Very often, a multitude of diverse risk factors can be present in a single patient, so the appropriate pharmacological protocol is very hard to find and use. All comorbidities recorded in our study are associated with adults of all ages, but the mortality rate and fatal outcome were more frequent in the adults who were older than 50 (30, 31). Comorbidities do not tell the full story about severity, form, and death risks in COVID-19 patients.

The second part of the study focused on the routine and specific laboratory blood tests. The laboratory blood test results were significantly different in the two groups (Table 2). The deceased patients had higher levels of serum creatinine, uremic acid, direct bilirubin, AST, LDH, CK, hsTnT, chloride ions, and C-reactive protein. On the other hand, the urea serum concentrations were significantly lower in the patients who survived (Table 2).

Regarding the total blood count and coagulation status, there were significant differences between the groups (Table 3). The higher counts of neutrophils, the higher concentration of D-dimer, and the higher activities of fibrinogen were recorded for the deceased patients. On the other hand, the deceased patients had lower levels of hemoglobin, platelets, lymphocytes, monocytes, and the Factor IX and Factor XII activity. (Table 3).

It has been recognized that one algorithm of laboratory markers could be a potential guide for making prognosis and developing useful therapeutical protocols. As a standard of care, the baseline blood tests and inflammatory markers are obtained when patients are admitted to a hospital. The proper approach to risk assessment should allow physicians to forecast a patient's future worsening based on these initial results. As it has been mentioned in the literature on numerous occasions, D-dimer is associated with a bad prognosis. Our findings confirm this. The seconds early diagnostic markers of hyper-coagulopathy are the disturbances in CRP, fibrinogen, and white blood cells. They can indicate the necessity to include direct factor Xa inhibitors in a treatment [29–31].

Elevated D-dimer level suggests extensive thrombin generation and fibrinolysis. It is associated with poor prognosis in COVID-19. This has prompted clinicians to hypothesize that increased D-dimer concentrations are indicative of coexisting venous thromboembolisms that may lead to ventilation-perfusion mismatch. Moreover, there is a lot of evidence about the high risk for thromboembolism in patients with COVID-19 disease. The activation of KKS in plasma leads to BK overproduction [32].

Moreover, it is essential to obtain the cut-off values for the severity of the disease for each laboratory marker in order to reduce the number of severe and fatal cases.

The secondary outcome for the two groups also differed (Table 4). The duration of the hospital treatment differed significantly in that the deceased patients who underwent longer in-hospital treatments (Table 4). Their CT scores were higher, they spent more time on the mechanical ventilation, and their ICU stays were longer. The time passing from the emergence of the first symptoms to the hospital admission was quite similar for the two groups. (Table 4). As expected, the ICU treatments were longer for the deceased patients. Chiam T et al. investigated the hospital length (LOS) among COVID-19-positive patients [33]. They concluded that COVID-19 patients' LOS varies based on multiple factors, such as older age, comorbidities, and disease severity. Deeper understanding of these factors is crucial in improving the accuracy of predictions about COVID-19 patients, which can further enhance resource and care planning and management in hospitals.

In conclusion, we tried to find the molecular mechanisms responsible for fatal outcomes of COVID-19 pneumonia. We detected the elevated oxidative stress and very high levels of serum bradykinin and galectin-3 at the patients' admission in those who passed away. Their levels of nitric oxide and the activity of superoxide dismutase were lower while the levels of superoxide anion radical and the indices of lipid peroxidation were higher (Figures 1–8). Bradykinin and galectin-3 concentrations were significantly higher in the deceased patients (Figures 5 and 6). Our results are in line with the results of the previous studies. Veerdonk et al. suggested that a bradykinin metabolite, des-Arg9-BK, could contribute to inflammation, vasodilation, and vascular permeability via the activation of bradykinin receptors [34]. Moreover, it is known that a possible source of the bradykinin in COVID-19 patients could be bronchiole and alveoli resident mast cells. It is well-known that, as tissue-resident granulocytes, mast cells can synthesize bradykinin via the secretion of heparin, the activation of coagulation factor XII, and the formation of plasma kallikrein. Therefore, the increase in bradykinin may occur due to the increased mast cell density in the lungs of COVID-19 patients [35, 36].

Using a correlation analysis, we confirmed there are certain relations between oxidative stress markers and cytokine bradykinin, but also peptide galectin-3 (Table 5). The serum bradykinin demonstrates a positive weak correlation with the levels of plasma hydrogen peroxide. On the other hand, there is an inverse weak correlation with the activity of superoxide dismutase. Galectin-3 levels correlate with the indices of lipid peroxidation in a similar manner (Table 5). Interestingly, we have detected the critical values of bradykinin in COVID-19 patients. The levels of serum bradykinin ranging from 200000 to 280000 pg/ml represent the borderline between the distribution of the survived and deceased patients. These values could be used as a significant diagnostic and prognostic sign for changing the therapy protocols and preventing fatal outcomes (Figures 6–8). On the other hand, galectin-3 does not exhibit any linear dynamics. Thus, it cannot be used as a sensitive diagnostic or prognostic marker.

Definitely, bradykinin storm was present in the deceased patients. Unfortunately, the ongoing storm can probably result in increased microvascular permeability, edema, and further inflammation, which worsen prognoses. Our study strongly supports the bradykinin storm hypothesis. In that sense, future therapeutical strategies must be focused on reducing a bradykinin serum concentration in COVID-19 patients. Studies conducted by Ghahestani et al. suggested that blocking the B2 receptor with icatibant may be a good strategy for large BK degradation in COVID-19 patients. As they concluded, this drug could be able to also reduce angioedema, improve oxygenation in severe forms of the disease, and prevent poor clinical outcomes in patients with COVID-19 pneumonia [37]. This phenomenon was described in the experimental models in which bradykinin and substance P were detected in very high concentrations in animals suffering from stroke and brain injuries [38–40].

Bradykinin can be a prognostic marker of mortality due to its linear dynamics and high sensitivity. At the heart of the pandemic, there was a growing concern about the pathophysiology of COVID-positive patients and their prognoses. Moreover, since there are no unique and effective pharmacological protocols for the patients infected with SARS-CoV-2, the importance of knowing the pathophysiological molecular mechanisms has been increasingly emphasized. The proinflammatory cytokines and peptides, such as bradykinin and galectin-3, are dominant in the deceased patients and may contribute to leaky vasculature and cell necrosis, which results in microvascular endotheliopathy. Moreover, a bradykinin course follows free radicals, which are identified in high bioavailability in the systemic circulation of patients with COVID-19 pneumonia. The cytokine and bradykinin storm theory could be a possible explanation for the severe forms of COVID-19 disease. As such, they offer many potential therapeutic targets for the prevention of multiorgan failure, such as inhibitors of bradykinin production [41–42].

The limitation of this study is its relatively small sample size. However, we included matched patients with similar clinical features and the duration of the disease prior to admission. Moreover, given the different pathophysiology of COVID-19 disease, we plan to include the selected patients with 7 or 28 day follow-ups who had a mild type of disease to test all these assumptions further.

## 5. Conclusion

Bradykinin storm and oxidative stress in patients with the fatal outcomes are probably responsible for the overactive inflammatory response and the resulting symptoms in COVID-19 patients. Understanding the mechanisms by which the two storms, bradykinin and cytokine, affect the body separately or jointly is crucial because it may help us develop more effective treatments, save lives, and mitigate the effects of the pandemic.

## Figures and Tables

**Table 1:** Demographic characteristics and comorbidity of COVID-19 patients at admission. Statistical significance was confirmed by Mann–Whitney *U* test or Chi Square Test.

Variables	Survived [*n* = 32]	Deceased [*n* = 15]	Statistical significance
Age [years], mean [SD]	46.50 ± 13.84	72.53 ± 9.95	*p* < 0.05∗
Gender, n [%] (M/F)	F 31.3% M 68.7%	F 26.7% M 73.3%	*p* < 0.05∗
Hypertension, *n* [%]	Yes 40.6%	Yes 93.3%	*p* < 0.05∗
Diabetes, *n* [%]	Yes 15.6%	Yes 20.0%	*p* < 0.05∗
Morbid obesity, *n* [%]	Yes 0%	Yes 6.67%	*p* < 0.05∗
Chronic obstructive pulmonary disease, *n* [%]	Yes 0%	Yes 0%	*p* > 0.05
Asthma, *n* [%]	Yes 0%	Yes 0%	*p* > 0.05
Coronary disease, *n* [%]	Yes 0%	Yes 6.67%	*p* < 0.05∗
Cardiomyopathy, *n* [%]	Yes 0%	Yes 6.67%	*p* < 0.05∗
Chronic kidney disease, *n* [%]	Yes 0%	Yes 6.67%	*p* < 0.05∗

**Table 2:** Laboratory blood test (liver and renal function and inflammatory markers) of COVID-19 patients at admission. Statistical significance was confirmed by Mann–Whitney *U* test.

Variables	Survived [*n* = 32]	Deceased [*n* = 15]	Statistical significance
Urea [mmol/l], mean [SD]	46.50 ± 13.84	10.55 ± 4.44	*p* < 0.05∗
Creatinine [mg/dl], mean [SD]	90.08 ± 15.21	124.46 ± 57.22	*p* < 0.05∗
Uremic acid [mg/dl], mean [SD]	271.92 ± 88.17	336.54 ± 158.26	*p* < 0.05∗
Glucose [mmol/l], mean [SD]	7.21 ± 3.01	8.06 ± 4.53	*p* > 0.05
Direct bilirubin [*μ*mol/l], mean [SD]	2.24 ± 0.68	4.23 ± 2.87	*p* < 0.05∗
Total bilirubin [*μ*mol/l], mean [SD]	6.47 ± 3.33	8.83 ± 6.16	*p* > 0.05
AST [U/l], mean [SD]	26.67 ± 13.67	53.00 ± 42.68	*p* < 0.05∗
ALT [U/l], mean [SD]	31.50 ± 26.86	31.77 ± 25.01	*p* > 0.05
ALP [U/l], mean [SD]	58.31 ± 13.20	61.38 ± 28.92	*p* > 0.05
LDH [U/l], mean [SD]	351.92 ± 74.89	734.77 ± 278.73	*p* < 0.05∗
CK [U/l], mean [SD]	125.21 ± 97.17	382.15 ± 365.28	*p* < 0.05∗
hsTnT [ng/ml], mean [SD]	6.96 ± 3.52	59.92 ± 12.23	*p* < 0.05∗
gamaGT [U/l], mean [SD]	104.08 ± 62.76	104.92 ± 22.12	*p* > 0.05
K [mmol/l], mean [SD]	4.33 ± 0.40	4.25 ± 0.61	*p* > 0.05
Na [mmol/l], mean [SD]	141.21 ± 1.47	140.23 ± 7.87	*p* > 0.05
Ca [mmol/l], mean [SD]	2.25 ± 0.18	2.08 ± 0.14	*p* > 0.05
P [mmol/l], mean [SD]	1.05 ± 0.21	1.09 ± 0.27	*p* > 0.05
Manganese [mmol/l], mean [SD]	0.84 ± 0.11	0.89 ± 0.18	*p* > 0.05
CL [mmol/l], mean [SD]	2.25 ± 0.18	100.52 ± 6.99	*p* < 0.05∗
Total proteins [g/l], mean [SD]	67.75 ± 6.43	69.92 ± 6.59	*p* > 0.05
Albumin [g/l], mean [SD]	42.50 ± 4.87	35.54 ± 4.77	*p* > 0.05
CRP [mg/l], mean [SD]	23.58 ± 33.08	167.64 ± 19.93	*p* < 0.05∗

**Table 3:** Total blood count and Coagulation status of COVID-19 patients at admission. Statistical significance was confirmed by Mann–Whitney *U* test.

Variables	Survived [*n* = 32]	Deceased [*n* = 15]	Statistical significance
Leu [x10^9^/l], mean [SD]	4.81 ± 1.30	5.52 ± 3.58	*p* > 0.05
Er [x10^12^/l], mean [SD]	4.67 ± 0.67	3.92 ± 0.77	*p* > 0.05
HGB [g/l], mean [SD]	136.50 ± 14.86	115.31 ± 23.12	*p* < 0.05^∗^
HCT [l/l], mean [SD]	0.41 ± 0.04	0.38 ± 0.11	*p* > 0.05
MCV [fl], mean [SD]	86.72 ± 3.41	88.75 ± 4.41	*p* > 0.05
TR [x10^9^/l], mean [SD]	209.38 ± 87.27	168.54 ± 69.23	*p* < 0.05^∗^
Neu [x103/*μ*l], mean [SD]	2.85 ± 0.95	4.29 ± 3.63	*p* < 0.001^∗∗^
Lym [x103/*μ*l], mean [SD]	1.33 ± 0.56	0.60 ± 0.35	*p* < 0.001^∗∗^
Mon [x103/*μ*l], mean [SD]	0.37 ± 0.23	0.14 ± 0.11	*p* < 0.05^∗^
INR, mean [SD]	0.94 ± 0.06	1.12 ± 0.47	*p* > 0.05
PT, mean [SD]	109.18 ± 8.85	93.58 ± 25.32	*p* > 0.05
aPTT [s], mean [SD]	26.33 ± 4.18	35.14 ± 14.73	*p* > 0.05
D-dimer [ng/ml], mean [SD]	355.50 ± 372.70	1460.23 ± 1135.17	*p* < 0.001^∗∗^
Factor II [ng/ml], mean [SD]	113.52 ± 17.00	93.31 ± 24.44	*p* > 0.05
Factor V [ng/ml], mean [SD]	121.80 ± 21.04	124.06 ± 18.37	*p* > 0.05
Factor VII [mg/ml], mean [SD]	119.88 ± 30.36	96.24 ± 29.31	*p* > 0.05
Factor VIII [ng/ml], mean [SD]	101.31 ± 36.23	113.01 ± 33.86	*p* > 0.05
Factor IX [ng/ml], mean [SD]	120.00 ± 20.19	98.65 ± 33.68	*p* < 0.05^∗^
Factor X [ng/ml], mean [SD]	111.60 ± 19.11	97.35 ± 33.17	*p* > 0.05
Factor XI [ng/ml], mean [SD]	102.69 ± 31.19	91.57 ± 29.76	*p* > 0.05
Factor XII [ng/ml], mean [SD]	114.20 ± 31.22	77.92 ± 22.66	*p* < 0.05^∗^
AT III [g/dl], mean [SD]	97.12 ± 12.22	81.02 ± 14.22	*p* > 0.05
Fibrinogen [g/l], mean [SD]	3.95 ± 1.06	5.09 ± 1.43	*p* < 0.05^∗^

**Table 4:** Secondary outcomes of COVID-19 patients. Statistical significance was confirmed by Chi Square Test.

Secondary outcomes	Survived [*n* = 32]	Deceased [*n* = 15]	Statistical significance
Duration from first symptoms to admission (days)	7.36 ± 4.15	6.31 ± 3.88	*p* > 0.05
Duration of hospital treatment (days)	7.46 ± 5.53	11.54 ± 4.27	*p* < 0.05^∗^
CT score	5.10 ± 3.23	20.30 ± 4.14	*p* < 0.05^∗^
Duration of invasive mechanical ventilation (days)	0.00	5.00 ± 3.81	*p* < 0.05^∗^
Duration of noninvasive mechanical ventilation (NIV) (days)	0.00	4.23 ± 2.95	*p* < 0.05^∗^
Duration in ICU (days)	0.00	8.54 ± 2.99	*p* < 0.05^∗^

**Table 5:** Correlation analysis between cytokines and parameters of redox balance in hospitalized patients with confirmed SARS-CoV-2 infection.

Variables	NO^−^	H_2_O_2_	O_2_-	TBARS	SOD	CAT	GSH
Bradykinin							
Pearson correlation coefficient	-0.007	0.332^∗^	0.259	0.282	-0.289^∗^	-0.122	0.142
*p* value	0.963	0.023	0.079	0.055	0.049	0.416	0.341
Galectin-3							
Pearson correlation coefficient	0.075	0.166	0.193	0.322^∗^	-0.049	-0.224	0.279
*p* value	0.623	0.277	0.204	0.031	0.749	0.140	0.063

**Table 6:** COX regression analysis of predictors of factors associated with mortality in patients with COVID-19 confirmed pneumonia.

Variables	Hazard ratio (95% CI)	*p* value	Adjusted hazard ration (95% CI)	*p* value
Age above 50 years	1.01 (1.00-1.02)	0.001^∗∗^	1.588 (1.131-2.227)	0.006^∗∗^
Duration from first symptom to admission	1.22 (1.17-1.26)	0.001^∗∗^	2.488 (1.865-3.432)	0.001^∗∗^
Creatinine > 1.5 mg/dl	1.47 (1.16-1.83)	0.002^∗∗^	0.835 (0.635-1.097)	0.187
D-dimer elevated	1.87 (1.32-2.55)	0.001^∗∗^	1.33 (0.857-1.722)	0.071
CRP elevated	1.63 (1.11-2.32)	0.001^∗∗^	1.45 (0.951-1.804)	0.152
Lymphocyte count elevated	0.74 (0.59-0.94)	0.011^∗^	0.531 (0.242-0.511)	0.001^∗∗^
Monocyte count decreased	1.01 (1.01-1.01)	0.001^∗∗^	1.12 (0.880-1.323)	0.259
Hypertension presence	2.33 (1.89-3.01)	0.001^∗∗^	1.801 (0.746-1.606)	0.072
Diabetes presence	1.45 (1.15-1.73)	0.001^∗∗^	0.935 (0.655-1.101)	0.134
Bradykinin above 200000 ng/ml	1.001 (1.001-1.001)	0.001^∗∗^	2.135 (1.666-2.567)	0.001^∗∗^
Galectine-3 above	1.07 (1.04-1.016)	0.001^∗∗^	1.182 (0.991-1.651)	0.244

## Data Availability

The unpublished data used to support the findings of this study are available from the corresponding author upon request.
